# The influence of colloidal parameters on the specific power absorption of PAA-coated magnetite nanoparticles

**DOI:** 10.1186/1556-276X-6-383

**Published:** 2011-05-16

**Authors:** Yolanda Piñeiro-Redondo, Manuel Bañobre-López, Iván Pardiñas-Blanco, Gerardo Goya, M Arturo López-Quintela, José Rivas

**Affiliations:** 1 Applied Physics and Physical Chemistry Departments, University of Santiago de Compostela, Santiago de Compostela, 15782, Spain; 2 R&D Department, Nanogap Subnmpowder SA, Milladoiro, Ames, A Coruña, 15985, Spain; 3 Instituto de Nanociencia de Aragón and Condensed Matter Physics Department, University of Zaragoza, Zaragoza, 50018, Spain

## Abstract

The suitability of magnetic nanoparticles (MNPs) to act as heat nano-sources by application of an alternating magnetic field has recently been studied due to their promising applications in biomedicine. The understanding of the magnetic relaxation mechanism in biocompatible nanoparticle systems is crucial in order to optimize the magnetic properties and maximize the specific absorption rate (SAR). With this aim, the SAR of magnetic dispersions containing superparamagnetic magnetite nanoparticles bio-coated with polyacrylic acid of an average particle size of ≈10 nm has been evaluated separately by changing colloidal parameters such as the MNP concentration and the viscosity of the solvent. A remarkable decrease of the SAR values with increasing particle concentration and solvent viscosity was found. These behaviours have been discussed on the basis of the magnetic relaxation mechanisms involved.

PACS: 80; 87; 87.85jf

## Introduction

Biocompatible magnetic nanoparticles (MNPs) are increasingly being used in many biomedical applications, such as magnetic resonance imaging, drug delivery, cell and tissue targeting or hyperthermia [1-3]. For hyperthermia therapy, nanotechnology offers a powerful tool to the design of nanometre heat-generating sources, which can be activated remotely by the application of an external alternating magnetic field (AMF). The magnetic energy absorption of nanoparticle-containing tissues induces a localized heating that allows a targeted cell death at a critical temperature above 42 to 45°C. This temperature increase can be used to selectively kill cancer cells [4,5]. Previous reports show that the effective use of MNPs to induce magnetic heating by application of an external radio-frequency magnetic field depends essentially on several factors related to the size, shape, solvent and magnetic properties of nanoparticles [6-9]. Of special interest is the heating power rate that can be attained with MNPs because an increase of the heating rate would imply lower doses of MNPs administered to the patient and lower time of stay in the body of the patient. For this reason, it is necessary to optimize the design of the nanoparticles in order to achieve the required structural and magnetic properties which lead to the maximum heating power.

For single-domain particles, which are below the superparamagnetic (SPM) size limit, no heating due to hysteresis losses occurs. Therefore, the heating power arises from the energy dissipated in the reversible process of relaxation of the magnetic moments to their equilibrium orientation once the magnetic field is removed. This mechanism is characterized by the Néel relaxation process. In addition to this, the rotational motion of the particles within the solvent due to the torque forces on the magnetic moment, Brownian relaxation, constitutes another source of heating, as a consequence of the energy liberated by friction in the reorientation of the particle in the surrounding carrier liquid. The well-known Rosensweig equation [10] predicts the SAR of a magnetic nanoparticle exposed to a varying magnetic field as SAR = *P*/(ρΦ), where *P *is the dissipated power heat:(1)

in which the magnetic susceptibility χ" contains the action of both relaxation mechanisms:(2)

Through an effective relaxation time of the two mechanisms working in parallel:(3)

where(4)

is the Brown relaxation time depending on the solvent viscosity η and the hydrodynamic radius of the NP, , and(5)

is the Néel relaxation time depending on the magnetic volume of the NP,  and K_an _is the magnetic anisotropy energy constant of the magnetic core of the NP.

Therefore, the heat dissipation of a magnetic hyperthermia experiment performed on a ferrofluid will depend on: (1) the applied magnetic field strength and frequency and (2) the physical properties of the ferrofluid: solvent viscosity, magnetic and hydrodynamic radius of the NPs, and the magnetic anisotropy energy constant of the magnetic core of the NP.

Adequately coated iron oxide-based nanoparticles have been the most extensively studied material in hyperthermia experiments because they have very low toxicity, making them suitable for in vivo applications [11,12]. In particular, the polyacrylic acid (PAA) coating is an aqueous soluble polymer with a high density of reactive functional groups which make it very attractive in biomedicine due mainly to its capability to form flexible polymer chain-protein complexes trough electrostatic, hydrogen bonding or hydrophobic interactions. Furthermore, the biochemical activity of the protein is maintained in the resulting protein-polymer complexes [13].

Therefore, the use of biocompatible SPM nanoparticles capable of residing inside the human body for a reasonable time is highly desirable for biomedical applications. The absence of coercive forces and remanence prevents the magnetic interaction between particles and the formation of particle aggregates and small clusters [1].

Both mechanisms depend on particle size, whereas only the Brownian contribution depends on the viscosity, η, of the carrier solvent. However, although the size dependence of the heating power has been already investigated and indicates the existence of an optimal particle size in which the heating power is maximum [14], there are no systematic data on the influence of particle concentration or solvent properties in the same magnetic system and in a simultaneous way. As deduced from the Rosensweig equation and under certain experimental conditions, both Néel and Brownian relaxation times are comparable for SPM nanoparticles around 10 nm; therefore, changes in the particle concentration, solvent viscosity or particle surface modification could lead to important differences in the SAR observed. To our best knowledge, no heating properties of PAA-modified high quality magnetite MNPs have been previously reported. Such combination of the chemical features described above makes colloidal PAA-magnetite a promising system in advanced bionanotechnologies. For this reason, data about its heating properties under specific experimental conditions, which could reproduce physiological conditions in an *in-vivo *experiment, are highly desired.

Our approach in this research includes the synthesis of different biocompatible and monodisperse high quality single-domain magnetite NPs based ferrofluids and has been focused on the specific absorption rate (SAR) dependence of factors related to the particle concentration and solvent properties, crucial parameters for the biomedical applications in order to provide the patients with an optimal dosage.

To our knowledge, we provide for the first time useful information in order to correctly interpret and design PAA-coated magnetite based biomedical applications in which the target tissues may have different viscosities and different capacity to retain low or high concentrations of NP inside, yielding unexpected results.

### Experimental

Iron oxide MNPs were obtained in order to study the effect of some colloidal parameters on their hyperthermia properties. Magnetite MNPs of ≈10 nm were synthesized by chemical co-precipitation of an aqueous solution containing Fe^2+ ^(FeSO_4_·7H_2_O, 99%) and Fe^3+ ^(FeCl_3_·6H_2_O, 97%) salts in the molar ratio Fe^2+^/Fe^3+ ^= 0.67 with ammonium hydroxide (NH_4_OH, 28%). To obtain Fe_3_O_4_@PAA MNPs, immediately after magnetite precipitation an excess of PAA (Mn = 1800) was added to the solution. The PAA coating reduces the electrostatic particle interactions and therefore greatly increases the colloidal stability of the dispersion. Finally, the pH of the solution was adjusted to pH = 10 by adding tetramethylammonium hydroxide (TMAOH) 10% in order to improve the stability of the ferrofluid as much as possible.

Specific absorption rate of the samples was measured by means of a home-made magnetic radio-frequency (RF) power generator operating at a fixed frequency of ν = 308 KHz and an induced magnetic field of *B *= 15 mT. A cylindrical Teflon sample holder was placed in the midpoint of an ethylene glycol cooled hollow coil (maximum of RF magnetic field), inside a thermally isolated cylindrical Dewar glass under high vacuum conditions (10^-6 ^mbar). Measurements were carried out by placing 140 μL of ferrofluid in the sample holder and recording the temperature increase versus time with a fibre-optic thermometer (Neoptix) during approx. 5 min of applied magnetic field.

## Results and discussion

The crystalline phase of iron oxide nanoparticles was identified by powder X-ray diffraction (XRD) using a PHILIPHS diffractometer with Cu Kα radiation λ = 1.5406 Å. The position and relative intensities of the reflection peaks confirm the presence of a magnetite/maghemite phase with espinel structure (JCPDS 19-0629). The crystallite size, *d*_(hkl)_, was calculated from the broadening (FWHM) of the (311) reflection following the Debye-Scherrer equation, resulting to be *d*_(311) _≈ 12 nm. It is important to remark that the absence of extra reflections indicated that no other iron oxides as secondary phases are present.

The attachment of the polymer to the magnetite particle surface was confirmed by far-transmission-infra-red (FTIR) spectroscopy using a Thermo Scientific-Nicolet 6700 spectroscope. Dried powder samples were measured directly using the attenuated total reflectance (ATR) option. The characteristic absorption frequencies of PAA related to the vibrational modes of the free carbonyl groups were identified in the PAA spectrum: C = O stretch at 1709 cm^-1^, C-O-H in-plane deformation at 1452 and 1415 cm^-1 ^and C-O stretch at 1250 cm^-1^. The position of these IR bands is in good agreement with previous experimental reported data [15]. After reaction between the PAA and magnetite NPs, a drastic intensity decreases of the C = O stretching peak at 1709 cm^-1 ^was observed. This strong intensity decrease of the C = O stretching peak and the appearance of new bands at 1547 and 1404 cm^-1^, which are due to the asymmetric and symmetric stretching of the COO^- ^carboxylate group, respectively, suggests that an efficient attachment between the polymer and the particle surface has taken place through the carbonyl group. By examining the frequency separation between the symmetric and the asymmetric COO^- ^stretching vibrations, Δν ≈ 150 cm^-1^, and taken into account the criteria established by Deacon and Phillips [16], the carboxylate group have been found to act as bridging complex. On the other hand, from thermogravimetric analysis (Perkin Elmer TGA 7 analyzer) the amount of PAA covering the magnetite nanoparticles was found to be 25% of the total mass. Taking these results into account, the estimated polymer shell thickness surrounding the magnetite NPs was around 1 nm.

**Figure 1:**
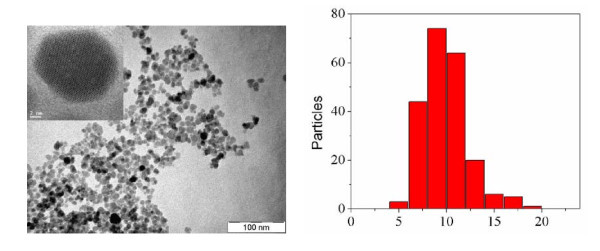
**(Left) TEM image of Fe**_**3**_**O**_**4**_**@PAA NPs**. Inset shows a brilliant field HR-STEM image of a single Fe_3_O_4_@PAA particle. (Right) Histogram corresponding to the Fe_3_O_4_@PAA NPs.

Morphology and crystal structure of PAA-coated magnetite nanoparticles were characterized by transmission electron microscopy (TEM) and scanning transmission electron microscopy (STEM) techniques using a PHILIPS CM-12 (100 kV) and a Hitachi S-5500 (30 kV) microscopes, respectively. Figure 1 (left) shows the uniform pseudo spherical shape of magnetite@PAA MNPs. The average particle size and distribution is shown in the corresponding histogram on the right and resulted to be highly monodisperse with *d *= 9 ± 2 nm (85% of the total amount of particles), in good agreement with the crystalline domain size calculated from XRD results. Inset of Figure 1 (left) shows a representative high-resolution (HR) brilliant field (BF) STEM micrograph of a single particle region, showing high crystallinity and the structural homogeneity of the particles. The long range domain structure and the absence of multi-domains suggest that these nanoparticles can be considered as small single crystals. It is also evidenced that the PAA coating prevents the formation of aggregates, since they are actually well separated from each other (as deduced from the distance between the whole particle in the middle of the picture and the surrounding ones shown at the edges).

**Figure 2:**
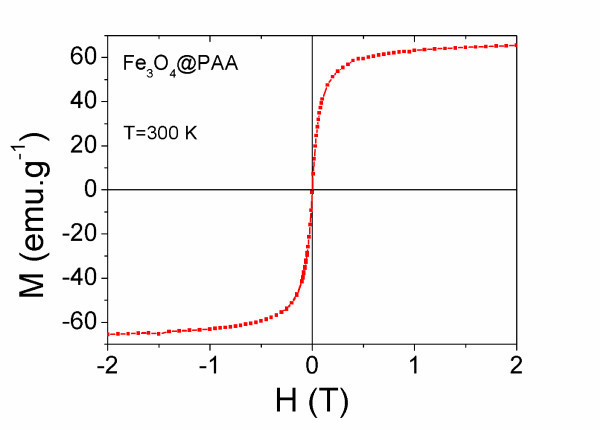
**Magnetization curves as a function of the applied magnetic field up to 2 T for Fe**_**3**_**O**_**4**_**@PAA NPs at room temperature**.

Figure 2 shows the magnetization curves as a function of the applied magnetic field up to 2 T for PAA-coated magnetite NPs performed in a superconducting quantum interference device (SQUID) magnetometer. A clear SPM behaviour is observed where coercive forces and remanence are elusive. This is in good concordance with the XRD and TEM/STEM results which evidenced that magnetite cores are within the size region below the single- to multi-domain limit, in which FM particles show a SPM-like behaviour. Magnetization of saturation, *M*_s_, is about 60 emu g^-1 ^at room temperature. However, after correction of the magnetic data by subtracting the non-magnetic mass corresponding to the PAA shell (that represents a 25% of the total mass, as deduced from the thermal analysis), the saturation increases again until 80 emu g^-1^, which is very close to the bulk magnetization for magnetite (90 emu g^-1^). This indicates that the intrinsic magnetic properties of the magnetite nuclei have not been affected by the coating.

### Magnetic hyperthermia results

The SAR for magnetic hyperthermia experiments has been calculated by using [14](6)

where *c*_liq _and *ρ*_liq _is the specific heat capacity and density of the liquid, respectively, and Φ the weight concentration of the MNPs in the colloid. By performing a linear fit of the hyperthermia data (temperature versus time) in the initial time interval, *t *= [1-10] s, we obtain the experimental value of . In this way, the SAR can be calculated using Equation 6, since all the remaining parameters are known.

#### Concentration effects

When the concentration of a ferrofluid is increased, the first obvious consequence is that the mean inter-particle distance is reduced. If the system is further exposed to an external RF magnetic field that magnetizes the SPM nanoparticles, magnetic dipolar interaction will become relevant and contribute to the magnetic properties of the ferrofluid. Since some controversies exists in theoretical studies about the influence of the dipolar interaction on the intrinsic magnetic properties of the MNPs [17], experimental measurements showing concentration effects on SAR properties of MNPs will help to elucidate the question.

**Figure 3:**
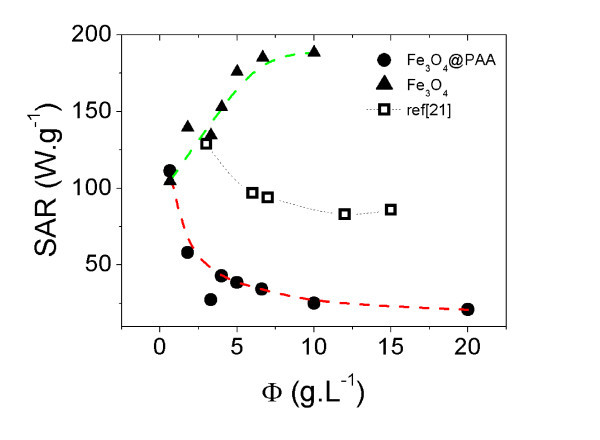
**Evolution of the specific absorption rate (SAR) of aqueous Fe**_**3**_**O**_**4**_**@PAA NPs dispersions at several concentrations between 0.6 and 20 g L**^**-1 **^**under an applied AC magnetic field of *B *= 15 mT and ν = 308 kHz**. Solid line is a guide for the eye.

In order to study the effect of the magnetite concentration on the hyperthermia properties of aqueous ferrofluids and to achieve an efficient temperature increase in the samples, we prepared two series of aqueous Fe_3_O_4 _and Fe_3_O_4_@PAA NPs based dispersions at different magnetite concentrations, ranging from 0.6 to 20 g L^-1^. Figure 3 shows the evolution of the SAR with magnetite concentration. The evolution of the SAR coefficient reveals that the heat production efficiency decreases with magnetite concentration for Fe_3_O_4_@PAA NPs, while a different behaviour is observed for bare Fe_3_O_4 _NPs. We associate this behaviour to the inter-particle dipole-dipole interactions, which are proportional to the particle concentration in the carrier fluid.

For Fe_3_O_4_@PAA NPs, as the particle concentration increases, particles get closer to each other increasing their dipolar magnetic moment interaction in presence of a RF external magnetic field. The energy dissipation mechanism directly involved and strongly dependent on the dipole-dipole interaction is the Néel relaxation time, since Brownian relaxation is much less sensitive to the concentration of magnetite moments because the inter-particle force is mainly hydrodynamic in nature [18]. The higher dipolar interactions the longer Néel relaxation times. Therefore, this long-range collective magnetic behaviour at increasing particle concentrations appears to play a major role in decreasing the SAR. In contrast, at very low particle concentrations the particles are more isolated from each other. In this scenario, the inter-particle dipolar interaction decreases dramatically with distance, ∝1/*r*^6^, and the efficiency of power dissipation to the medium is highly optimized. Although similar results have been previously reported in the literature in other magnetic systems, there are few works dealing with the effects of magnetic interactions on SAR, being mostly not comparable or controversial: Urtizberea et al. [19] showed a SAR increase with dilution of ≈11 nm maghemita nanoparticles based ferrofluids, although the study was carried out through AC susceptibility measurements performed below ≈100 kHz; and while [20] reported a higher SAR for tightly associated dextran-coated iron oxide nanoparticles (*d *≈ 90 nm) than for a more loosely associated ones, in [9], no concentration effects were detected. Figure 3 includes experimental data from Linh et al. [21] for relatively comparable colloidal magnetite based ferrofluid. A similar SAR dependence of the particle concentration is observed, although differences in the absolute values could derived from the slightly different particle size, particle distribution, coating agent or experimental conditions of frequency and applied magnetic field. It is important to mention that such a similar concentration heating efficiency was also observed in a different than magnetite system based on Ni-Zn ferrite nanoparticles dispersed in a shape memory polymer [22].

In the opposite, the SAR behaviour of bare Fe_3_O_4 _NPs is completely different. From the obtained results, we deduce that the differences observed in the SAR dependence of the particle concentration between the bare and PAA coated particles can be attributed to the active role played by the PAA shell. The PAA coating not only stabilizes the SPM nanoparticles in the aqueous medium mediating the inter-particle dipolar interaction (directly related to the Néel relaxation time), but also changes the hydrodynamic radius of the particles and modify the Brownian relaxation time by friction of the nanoparticle surface in the carrier fluid. In the case of bare magnetite nanoparticles, significant dipolar interactions are still present at low particle concentrations, while aggregation phenomena and cluster formation occurs at high particle concentrations. However, further work is needed in order to address in more detail this issue. A similar behaviour has been also reported by Verges et al. [23] for higher magnetite particle sizes, although the SAR values are significantly lower.

#### Solvent viscosity effect

In order to evaluate separately the Brownian contribution to the general hyperthermia mechanism in SPM magnetite nanoparticles, the heating properties of magnetic dispersions at a fixed particle concentration have been evaluated as a function of the solvent viscosity, η, which is directly related to the Brownian relaxation through Equation 4. In the presence of an AMF, the MNP will rotate trying to align its magnetic dipolar moment to the direction of the magnetic field. The friction of the particle with the solvent will generate heat and this mechanism is known as Brownian relaxation. It contributes to the total heating in competence with the Néel relaxation, in which the magnetic moment of the particle reorients internally without the physical rotation of the particle. Brown relaxation time increases with NP size and solvent viscosity giving rise to an increase in SAR values. However, when τ_B _becomes too much high τ_eff _= τ_Néel _and only Néel relaxation contributes to the heat dissipation mechanism. Therefore, for very viscous solvents, the Brownian contribution is blocked and only Néel relaxation contributes, decreasing the SAR.

**Figure 4:**
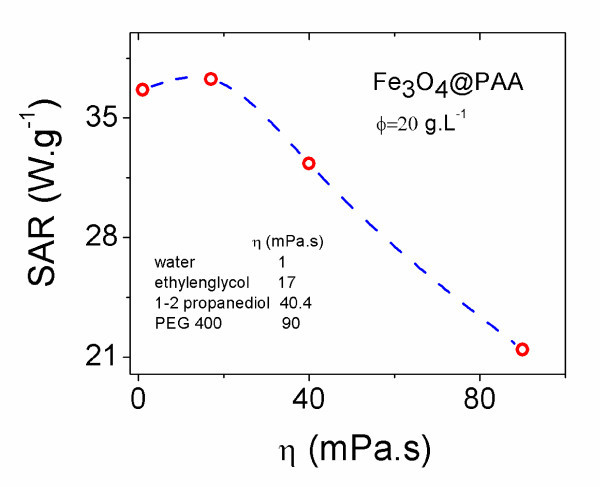
**Evolution of the specific absorption rate (SAR) of Fe**_**3**_**O**_**4**_**@PAA NPs dispersions with solvent viscosity, η, under an external AC magnetic field of *B *= 15 mT and ν = 308 kHz**. Solid line is a guide for the eye.

Figure 4 shows the evolution of SAR for PAA-coated magnetite ferrofluids with viscosity. Different values of viscosity ranging from 1 to 90 mPa s were achieved by using different solvents (water, ethylene glycol, 1-2-propanediol and poly-ethylene glycol). It is important to mention that the magnetite concentration was kept constant in all the samples, which showed a very good stability for all the solvents used. The effect of changing the solvent viscosity reveals that Brownian relaxation contribution is also significant in small SPM nanoparticles. A slight SAR increase from 36.5 to 37.3 W g^-1 ^takes place as the solvent viscosity increases from η = 1 mP s (water) to η = 17 mP s (ethylene glycol). However, the use of solvents of higher viscosities causes significant SAR decreases. This tendency agrees with theoretical predictions [10] and experimental results found in dextran-coated magnetite ferrofluids, where a maximum SAR is observed in the interval of 1 < η < 3 mP s [24].

The maximum of heat dissipation occurs for Equation 1 when the mathematical condition 2π*f*τ_eff _= 1 is fulfilled [6]. Therefore, concerning viscosity, the maximum will be observed for a certain value:(7)

If one changes experimental conditions involved in Equation 7 (particle size, strength and frequency of applied magnetic field, coating agent or magnetic material of the NPs), the location, height and width of the maximum of heat dissipation curve can change completely, giving rise to a variety of magnetic SAR relationships with viscosity. This explains why in literature one can find different behaviours of SAR with viscosity: a viscosity independent curve, a decaying one or even an increasing one, just only by varying the particle sizes and composition [25]. Also a Lorentzian curve, with a maximum located at certain values of viscosity, has been reported [24].

In this sense, the maximum of our SAR curve is obtained for a higher viscosity value than [19] because the chemical/physical characteristics of our MNPs (size, morphology, coating, etc.) and the experimental conditions of the applied RF magnetic field are different.

## Conclusions

Biocompatible PAA-coated magnetite based ferrofluids containing SPM nanoparticles of ≈10 nm have been chemically synthesized. The influence of several colloidal parameters on the specific power absorption of these magnetic dispersions has been studied. Particle concentration dependence of SAR has been mainly observed at low magnetite concentrations and a maximum in the SAR has been suggested as a function of the solvent viscosity around 22 mPa s.

## Abbreviations

ATR: attenuated transmission reflectance; BF: brilliant field; FTIR: far transmission infra-red; HR: high resolution; MNPs: magnetic nanoparticles; Ms: magnetization of saturation; PAA: poly(acrylic acid); RF: radio frequency; SAR: specific absorption rate; SPA: specific power absorption; SPM: superparamagnetic; STEM: scanning transmission electron microscopy; SQUID: superconducting quantum interference device; TEM: transmission electron microscopy; TMAOH: tetramethylammonium hydroxide; XRD: X-ray diffraction.

## Competing interests

The authors declare that they have no competing interests.

## Authors' contributions

YP-R carried out the hyperthermia/SAR measurements, participated in the discussion and helped to draft the manuscript. MB-L participated in the design of the study, in the synthesis and chemical/physical characterization of the samples, in the discussion and drafted the manuscript. IP-B participated in the synthesis and chemical characterization of the samples. GG was involved in the design and fabrication of the hyperthermia equipment, participated in the discussion and revised the manuscript. MAL-Q participated in the discussion and revised the manuscript. JR participated in its design, coordination and revised the manuscript. All the authors read and approved the final manuscript.
